# Ultrasound findings of diffuse metastasis of gastric signet-ring-cell carcinoma to the thyroid gland

**DOI:** 10.1007/s10396-016-0746-5

**Published:** 2016-10-01

**Authors:** Koji Morita, Takahiko Sakamoto, Shuji Ota, Hideo Masugi, Ikumi Chikuta, Yamato Mashimo, Naoki Edo, Takuo Tokairin, Nobuhiko Seki, Toshio Ishikawa

**Affiliations:** 1 Division of Endocrinology and Metabolism, Department of Internal Medicine, Teikyo University School of Medicine, 2-11-1 Kaga, Itabashi-ku, Tokyo, 173-8606 Japan; 2 Division of Medical Oncology, Department of Internal Medicine, Teikyo University School of Medicine, Itabashi-ku, Tokyo, Japan; 3 Department of Laboratory Medicine, Teikyo University School of Medicine, Itabashi-ku, Tokyo, Japan; 4 Department of Pathology, Teikyo University School of Medicine, Itabashi-ku, Tokyo, Japan

**Keywords:** Thyroid gland, Neoplasm metastasis, Stomach neoplasm, Signet-ring-cell carcinoma, Color Doppler ultrasonography

## Abstract

It has been shown that metastases to the thyroid from extrathyroidal malignancies occur as solitary or multiple nodules, or may involve the whole thyroid gland diffusely. However, diffuse metastasis of gastric cancer to the thyroid is extremely rare. Here, we report a case of a 74-year-old woman with diffuse infiltration of gastric adenocarcinoma (signet-ring-cell carcinoma/poorly differentiated adenocarcinoma) cells in the thyroid. The pathological diagnosis was made based on upper gastrointestinal endoscopy with biopsy and fine-needle aspiration cytology of the thyroid. An 18F-FDG PET/CT revealed multiple lesions with increased uptake, including the bilateral thyroid gland. On thyroid ultrasound examination, diffuse enlargement with internal heterogeneity and hypoechoic reticular lines was observed. On color Doppler imaging, a blood-flow signal was not detected in these hypoechoic lines. These findings were similar to those of diffuse metastases caused by other primary cancers, such as lung cancer, as reported earlier. Therefore, the presence of hypoechoic reticular lines without blood-flow signals is probably common to diffuse thyroid metastasis from any origin and an important diagnostic finding. This is the first report to show detailed ultrasound findings of diffuse gastric cancer metastasis to the thyroid gland using color Doppler.

## Introduction

Metastatic cancer to the thyroid, which is rarely diagnosed during life [1], either forms nodules or spreads diffusely in the thyroid [2]. The most common type is solitary or multiple nodules originating from hematogenous metastasis of renal cell cancer [3, 4]. On the other hand, lung cancer or other extrathyroidal malignancies may metastasize to the whole thyroid gland diffusely without forming nodules, although such cases are extremely rare [5]. Therefore, the enlarged thyroid gland due to diffuse thyroid metastasis might be misrecognized simply as autoimmune thyroiditis or adenomatous goiter, both of which are so common that they can happen in tumor-bearing patients as well.

The pathophysiology of diffuse thyroid metastasis has not been completely elucidated. In the cases of diffusely metastasized malignancies to the thyroid gland, advanced lymph node metastases are often observed, suggesting the involvement of lymphatic vessels [2, 6]. In addition, there was a case report, in which chylous, presumably lymphatic, and fluid were aspirated from the enlarged thyroid that was diffusely infiltrated by lung adenocarcinoma cells [2]. Therefore, lymphatic dissemination followed by microembolization is implicated in the process of diffuse thyroid metastasis [2]. In addition, the type of primary cancer may be an important factor in the development of diffuse thyroid metastasis. For example, even though lymphatic as well as hematogenous metastasis of gastric cancer is frequently seen, the probability of its metastasis to the thyroid seems very low [6–13], and there is only one case report of diffuse thyroid metastasis from gastric carcinoma [6], in particular, in the PubMed database.

We here present a case with diffuse metastasis from gastric cancer (signet-ring-cell carcinoma/poorly differentiated adenocarcinoma) to the thyroid gland. In this patient, we had a chance to perform thyroid color Doppler sonography, which was not carried out in the above-mentioned previous report on a similar patient [6]. Thus, the ultrasonographic findings obtained in this case are definitely valuable. Our report has revealed that the characteristic ultrasonographic features seen in our patient are analogous to those observed in diffuse thyroid metastases from other primary cancers [2, 14], and that color Doppler ultrasonography may be quite useful for the correct diagnosis of diffuse thyroid infiltration of gastric and other cancers.

## Case presentation

**Fig. 1:**
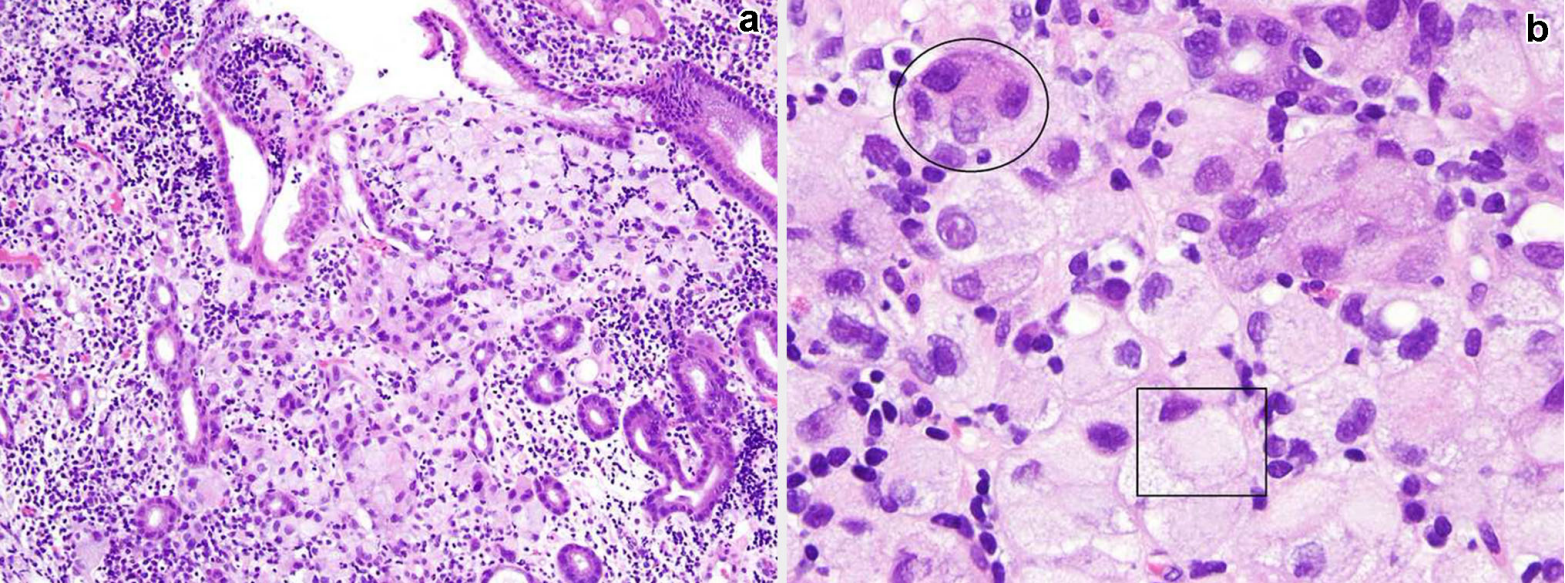
Histopathology of the primary gastric tumor. **a** Hematoxylin and eosin stain; original magnification ×100. Atypical cells with cytoplasmic mucin are diffusely invading the gastric mucosa. **b** Hematoxylin and eosin stain; original magnification ×400. *Round-shaped* cells with cytoplasmic mucin vacuoles and eccentrically placed nuclei are components of signet-ring-cell carcinoma (*rectangle*). Cells with a high nuclear-to-cytoplasmic ratio are components of poorly differentiated adenocarcinoma (*oval*)

**Fig. 2:**
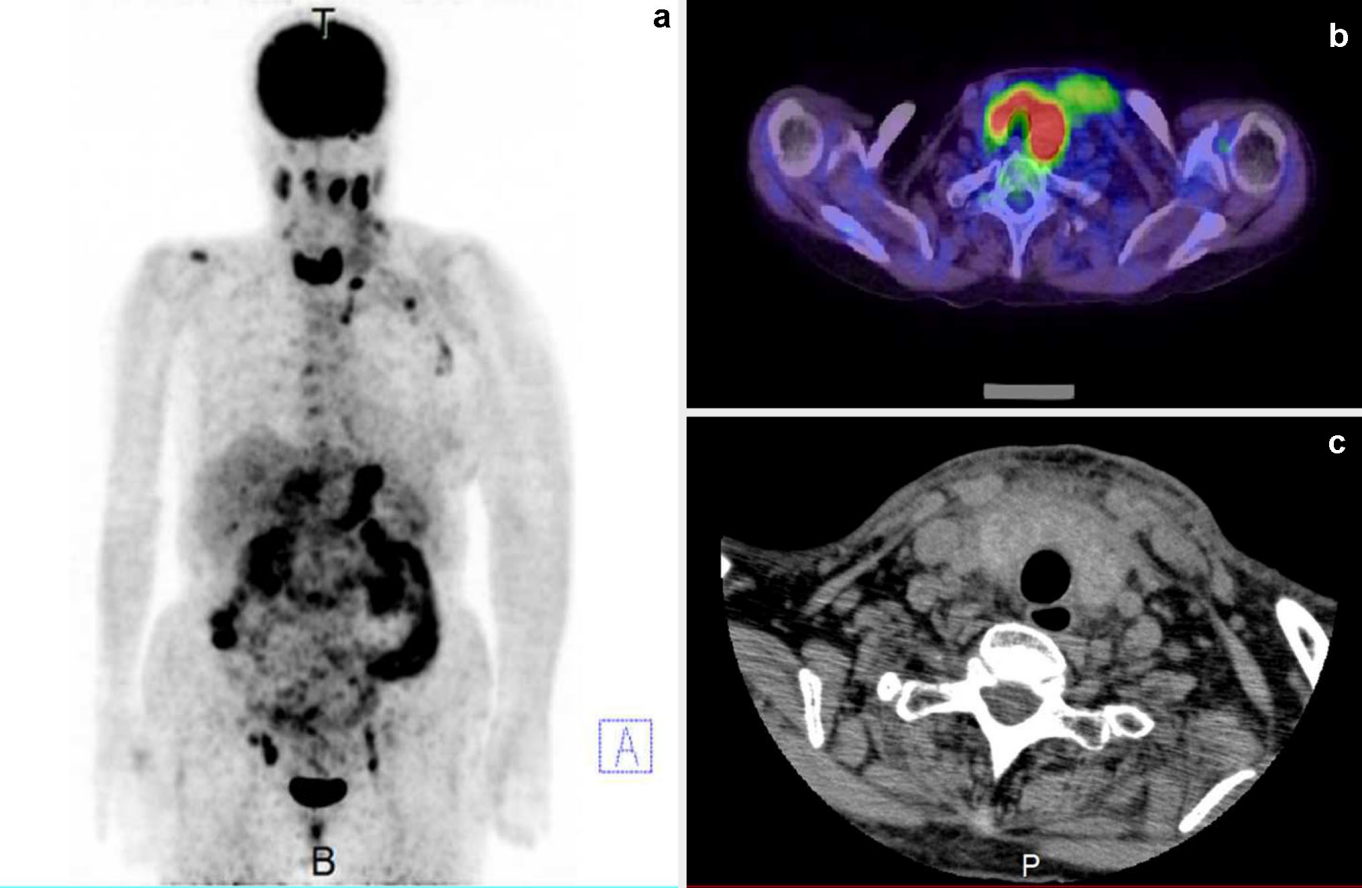
**a** Coronal maximum intensity projection (MIP) of 18F-FDG PET imaging before admission. Accumulation was found in the stomach, in the right hepatic lobe, in the extensive lymph node metastases, and in the whole thyroid gland. **b** Transverse section of the thyroid on 18F-FDG PET/CT imaging before admission. Diffuse uptake in bilateral thyroid lobes was observed. **c** Transverse section of the thyroid on CT imaging after admission. The thyroid gland was diffusely swollen. Its size enlarged and its CT value decreased after hospitalization. In addition, the adipose tissue concentration in the surrounding area increased

The patient was a 74-year-old female. Edema appeared in the upper left limb 6 weeks prior to hospitalization. A computed tomography (CT) scan revealed a thrombus in the left subclavian vein with adjacent lymphadenopathy. Therefore, anticoagulant therapy was initiated. The CT scan also showed the presence of ascites and wall thickening of the greater curvature of the gastric corpus, which prompted us to perform upper gastrointestinal endoscopy. The tumor found in the greater curvature of the middle body in the stomach was biopsied and diagnosed as adenocarcinoma (signet-ring-cell carcinoma/poorly differentiated adenocarcinoma; Fig. 1). On 2-deoxy-2-(18F) fluoro-d-glucose positron emission tomography/computed tomography imaging (PET/CT), accumulation was detected in the entire stomach; in the portal and posterior area of the right hepatic lobe; in the cervical, left supraclavicular, left axillary, left parasternal, and superior mediastinal lymph nodes (which suggested extensive lymph node metastases); and interestingly and unexpectedly, in the thyroid, where 18F-FDG was diffusely taken up in both lobes, for some unknown reason (Fig. 2a, b). The patient was diagnosed with multiple metastases of gastric signet-ring-cell carcinoma/poorly differentiated adenocarcinoma, and was subsequently admitted to our hospital for chemotherapy. Serum concentrations of carcinoembryonic antigen (CEA), CA19–9, and CA125 were elevated (9.7 ng/mL, 1825.0, and 122.5 U/mL, respectively), consistent with advanced gastric carcinoma. Thyroid gland function was within the standard range. Serum thyroglobulin was supranormal (893 ng/mL). Anti-thyroglobulin and anti-TPO antibodies were negative (Table 1).

**Table 1:** Laboratory results of the present case at the time of admission

WBC (/μL)	5800	Corrected calcium (mg/dL)	9.6
Hb (g/dL)	12.5	Total cholesterol (mg/dL)	146
Platelet count (×10^4^/μL)	21.0	Triglyceride (mg/dL)	89
		HDL-cholesterol (mg/dL)	37
TP (g/dL)	6.1	Plasma glucose (mg/dL)	107
ALB (g/dL)	2.9	C-reactive protein (mg/dL)	1.15
AST (U/L)	19		
ALT (U/L)	12	CEA (ng/mL)	9.7
LDH (U/L)	272	CA19–9 (U/mL)	1825
γ-GTP (U/L)	25	CA125 (U/mL)	122.5
CK (U/L)	69		
BUN (mg/dL)	12.4	TSH (μIU/mL)	1.35
Creatinine (mg/dL)	0.59	F-T4 (ng/dL)	1.51
Uric acid (mg/dL)	3.7	F-T3 (pg/mL)	2.47
Sodium (mEq/L)	143	Thyroglobulin (ng/mL)	843
Potassium (mEq/L)	3.6	Anti-thyroglobulin antibody (IU/mL)	15
Chloride (mEq/L)	108	Anti-thyroid peroxidase antibody (IU/mL)	<5

*WBC* white blood cell count, *Hb* hemoglobin, *TP* total protein, *ALB* serum albumin, *AST* aspartate aminotransferase, *ALT* alanine aminotransferase, *LDH* lactate dehydrogenase, *γ-GTP* γ-glutamyltransferase, *CK* Creatine phosphokinase, *BUN* blood urea nitrogen, *CEA* carcinoembryonic antigen, *CA19–9* carbohydrate antigen 19–9, *CA125* cancer antigen 125, *TSH* thyroid-stimulating hormone, *F-T4* free thyroxine, *F-T3* free triiodothyronine

On day 8 of hospitalization, the patient complained of bilateral neck swelling with pain on the left side. A CT scan was ordered, and it showed edema of the chin, neck, and anterior chest and increased density of adipose tissue. As for the thyroid, both the lobes were diffusely enlarged and exhibited low density on CT (Fig. 2c). Because PET revealed that a significant accumulation in the whole thyroid and antithyroid autoantibodies was negative, we suspected diffuse infiltration of gastric carcinoma cells into the thyroid. Therefore, ultrasound examination and aspiration cytology were performed.

**Fig. 3:**
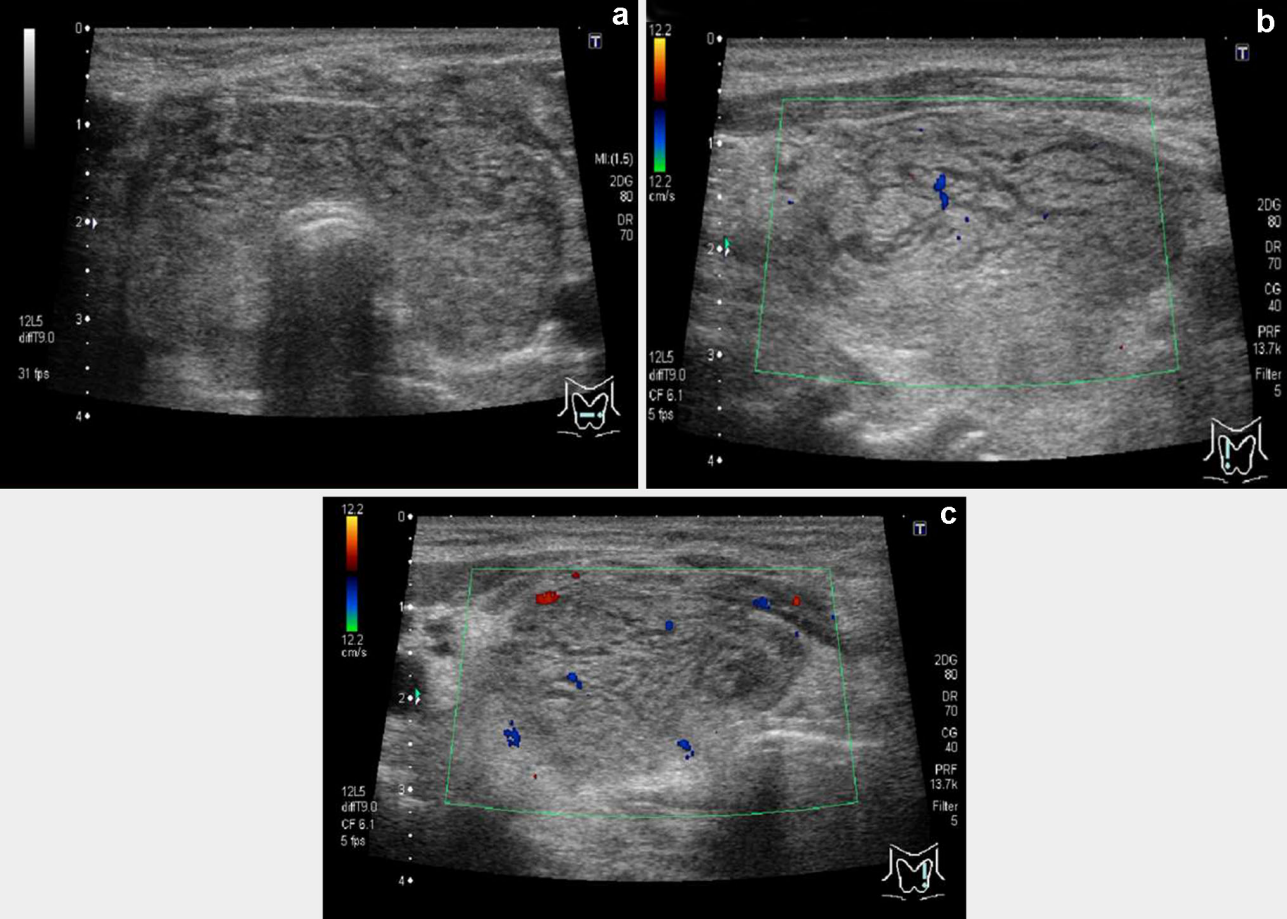
Thyroid ultrasonography after admission. **a** Transverse section of the thyroid gland on B-mode (brightness mode) ultrasound imaging. The thyroid was diffusely enlarged, with a 10-mm-thick isthmus. No nodular lesion was observed. The thyroid parenchyma was not of uniform echogenicity, with many hypoechoic reticular lines scattered in it. **b** Longitudinal section of the right lobe of the thyroid on color Doppler ultrasound imaging. **c** Longitudinal section of the left lobe of the thyroid on color Doppler ultrasound imaging. A Doppler signal was not detected in the hypoechoic reticular lines

**Fig. 4:**
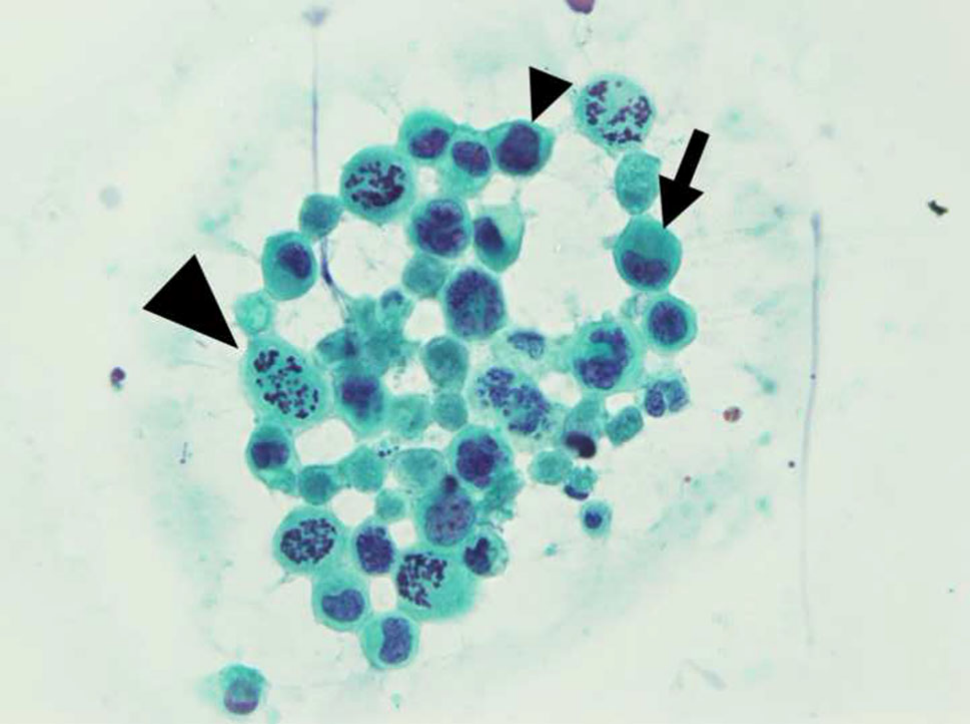
Cytology specimen that was obtained by fine-needle aspiration from the right lobe of the thyroid gland (Papanicolaou stain; original magnification ×400). Discohesive atypical cells with irregular hyperchromatic nuclei containing prominent nucleoli were present. Round-shaped cells with cytoplasmic mucin vacuoles and eccentrically placed nuclei were signet-ring-cell carcinoma cells (*arrow*). Cells with a high nuclear-to-cytoplasmic ratio were thought to be poorly differentiated adenocarcinoma cells (*arrowhead*). There were numerous mitotic figures (*big arrowhead*). Based on these findings, the thyroid lesion was defined as “malignant” (metastatic carcinoma) by TBSRTC

On ultrasonography, diffuse enlargement of the thyroid gland was seen (right lobe, 51 × 25 × 26 mm; left lobe, 49 × 51 × 20 mm; thickness of the isthmus, 10 mm), and it appeared internally heterogeneous. Nodular lesions were not detected, and hypoechoic reticular lines were observed in some places. On color Doppler imaging, the blood-flow signal was low (Fig. 3: imaging by Aplio-XG; TOSHIBA Medical Systems Corporation). The fine-needle aspiration cytology sample obtained from the right lobe revealed discohesive cells with severe nuclear atypia. Notably, there were cells with large cytoplasmic mucin vacuoles and cells with a high nuclear-to-cytoplasmic ratio. These findings indicated that the thyroid lesion was categorized as “malignant” (metastatic carcinoma) according to the Bethesda system for reporting thyroid cytopathology (TBSRTC) [15], and were consistent with the metastasis of the gastric signet-ring-cell carcinoma/poorly differentiated adenocarcinoma to the thyroid gland (Fig. 4). In addition, skin biopsy from the swollen anterior chest revealed insular tumor cells in the dermis, small blood vessels, and lymphatics. The vascular and lymphatic microembolizations caused by tumor cells established the diagnosis of cutaneous metastasis. Subsequently, the patient’s respiratory tract edema worsened, as confirmed by laryngoscopy, and glucocorticoids were administered. Chemotherapy with paclitaxel was done only once, because severe cytopenia occurred and her performance status became Eastern Cooperative Oncology Group (ECOG) class 4. Despite our best supportive care, the patient died a month after hospitalization. An autopsy was not performed.

## Discussion

The prevalence of metastatic thyroid gland cancer has been reported as 3.9 % by Mortensen et al. (18 out of 467 cases) [16] and 1.9 % by Abrams et al. (19 out of 1000 cases) [17], indicating that this is not a completely uncommon occurrence. However, endocrinologists and endocrine surgeons, who regularly treat thyroid diseases, hardly ever come across this disorder in clinical practice. For example, according to a report by the Mayo Clinic, of 20,262 surgical cases involving the thyroid gland, there were only 10 cases (0.05 %) of cancer metastasis to the thyroid [1]. As for the primary malignancy of thyroid metastasis, breast, lung, and colon cancers are usually found in autopsy cases [17], whereas kidney cancer is the most common, followed by breast, lung, and colon cancer, in live patients [3, 4]. Overall, however, metastasis to the thyroid is rarely encountered clinically, possibly because malignant cells may not be able to easily settle down and form colonies in the thyroid, which is a unique environment with its abundant arterial blood flow and its high oxygen and iodine content [4].

Among the rare cases with metastatic lesions in the thyroid, metastasis from gastric adenocarcinoma is even rarer, and to the best of our knowledge, there are only eight case reports in the PubMed database [6–13] (Table 2). In such cases, the gastric cancer is usually poorly differentiated, with multiple metastases in different organs at the time of diagnosis. The mean survival period has been reported to be approximately 5 months [6–13].

**Table 2:** Reported cases of metastatic thyroid tumor from gastric cancer (listed in chronological order)

Age/gender (References)	Pathology	Thyroid function	Thyroid ultrasound findings	Treatment	Survival (months)
71/M [7]	Poorly	Euthyrioidism (only serum T3 level was decreased)	Undescribed (a CT scan revealed that the tumor occupied almost the entire thyroid gland and extended to the mediastinum)	Bilateral subtotal thyroidectomy and radiotherapy	7
60/F [8]	Poorly	Euthyroidism	4 × 5-cm solid mass in the right lobe and two cystic masses, 1.5 and 2.5 cm in diameter, respectively, in the left lobe	Bilateral subtotal thyroidectomy	1
39/F [9]	Adenocarcinoma	Undescribed	Undescribed	None	1
63/F [6]	Signet-ring, poorly	Undescribed	Diffuse nodular enlargement of both lobes	Chemotherapy	6
71/M [10]	Poorly	Undescribed	Undescribed	Bilateral total thyroidectomy	4
68/M [11]	Signet-ring, poorly	Thyrotoxicosis	3.1 cm-sized tumor in the left lobe, which showed mosaic echogenicity and no calcification inside with a partially unclear border but no apparent spicular formation	None	1
67/M [12]	Signet-ring	Euthyroidism	A heterogeneous lobulated mass in the right lobe	Thyroidectomy and chemotherapy	Alive (14 months)
58/M [13]	Poorly	Euthyroidism	3 × 3 × 6-cm solid mass in the right lobe	Radiotherapy	5
74/F (present case)	Signet-ring, poorly	Euthyroidism	Diffusely enlarged heterogeneous thyroid with hypoechoic reticular lines	Chemotherapy	1

*Poorly* poorly differentiated adenocarcinoma, *Signet-ring* signet-ring-cell carcinoma

In our patient, thyroid gland ultrasound examination was performed, while she was alive, and characteristic findings were detected; instead of nodular lesions that are typically observed with metastatic tumors, diffuse changes were seen in the thyroid parenchyma. Ultimately, together with the PET findings and the cytological examination, we made a diagnosis of metastatic gastric cancer to the thyroid gland. Diffusely metastatic cancer to the thyroid is relatively rare, comprising 6 % of all intrathyroidal metastases [5], and is reported to originate from the lung, bile duct, penis, and stomach cancers [2, 6, 14, 18]. Kim et al. summarized ultrasonographic characteristics of 13 cases with diffuse metastasis to the thyroid (9 lung cancers, 2 unknown primary cancers, 1 cholangiocarcinoma, and 1 penile cancer) [14]. They reported that in these cases, the echogenicity of the enlarged thyroid gland was heterogeneously hypoechoic or isoechoic, and that internal hypoechoic lines were observed without increased vascularity on power Doppler ultrasonography [14]. We saw similar ultrasound findings in our patient, suggesting that these hypoechoic reticular lines without blood-flow signals may be characteristic of diffuse metastases to the thyroid, regardless of the origin of the primary malignancy. Because histopathological assessment of the thyroid was not performed either in the cases reported by Kim et al. or in our patient, the exact histological origin of the hypoechoic reticular lines has not been identified. However, the absence of blood-flow signals indicates that this image may represent intrathyroidal lymphatics dilated and packed with cancer cells. This possibility is quite likely, in light of concomitant metastatic lymphadenopathy involving cervical lymph nodes (Fig. 2a) and lymphatic tumor emboli in the surrounding skin observed in our case. Although 18F-FDG imaging is generally performed for the detection of metastases in cancer-bearing patients, diffuse accumulation of 18F-FDG may be seen even in the thyroid with no metastatic lesions as well, due to the coincidental presence of autoimmune thyroid disease (Hashimoto’s thyroiditis and Graves’ disease) [19], which is very common in the general population. In such situations, as was demonstrated in our case, ultrasonography is helpful to differentiate autoimmune thyroid diseases and diffuse cancerous cell infiltration. In Hashimoto’s thyroiditis, uneven echogenicity in the enlarged thyroid parenchyma is observed, but vessel-like hypoechoic linear structures are generally absent. In Graves’ disease, accelerated blood flow on color Doppler sonography is characteristic. Thus, hypoechoic reticular lines without increased blood flow may be highly diagnostic of diffuse cancer metastasis to the thyroid.

## Conclusions

We have presented a rare case of gastric signet-ring-cell carcinoma/poorly differentiated adenocarcinoma with diffuse metastasis in the thyroid gland. As was reported previously, hypoechoic reticular lines without blood-flow signals were observed on ultrasonography, which might be of high diagnostic value. Thus, ultrasound examination with color Doppler is thought to be very effective in detecting diffuse as well as nodular metastasis of malignant cells, including gastric cancer cells, to the thyroid.
